# Organ-Specific Differential NMR-Based Metabonomic Analysis of Soybean [*Glycine max* (L.) Merr.] Fruit Reveals the Metabolic Shifts and Potential Protection Mechanisms Involved in Field Mold Infection

**DOI:** 10.3389/fpls.2017.00508

**Published:** 2017-04-25

**Authors:** Jun-cai Deng, Cai-qiong Yang, Jing Zhang, Qing Zhang, Feng Yang, Wen-yu Yang, Jiang Liu

**Affiliations:** ^1^Key Laboratory of Crop Ecophysiology and Farming System in Southwest China, Ministry of AgricultureChengdu, China; ^2^Sichuan Engineering Research Center for Crop Strip Intercropping SystemChengdu, China; ^3^Institute of Ecological Agriculture, Sichuan Agricultural UniversityChengdu, China

**Keywords:** soybean, field mold, NMR, metabonomics, organ specificity

## Abstract

Prolonged, continuous rainfall is the main climatic characteristic of autumn in Southwest China, and it has been found to cause mildew outbreaks in pre-harvest soybean fields. Low temperature and humidity (LTH) stress during soybean maturation in the field promotes pre-harvest mildew, resulting in damage to different organs of soybean fruits to different extents, but relatively little information on the resistance mechanisms in these fruits is available. Therefore, to understand the metabolic responses of soybean fruits to field mold (FM), the metabonomic variations induced by LTH were characterized using proton nuclear magnetic resonance spectroscopy (^1^H-NMR), and the primary metabolites from the pod, seed coat and cotyledon of pre-harvest soybean were quantified. Analysis of FM-damaged soybean germplasms with different degrees of resistance to FM showed that extracts were dominated by 66 primary metabolites, including amino acids, organic acids and sugars. Each tissue had a characteristic metabolic profile, indicating that the metabolism of proline in the cotyledon, lysine in the seed coat, and sulfur in the pod play important roles in FM resistance. The primary-secondary metabolism interface and its potential contribution to FM resistance was investigated by targeted analyses of secondary metabolites. Both the seed coat and the pod have distinct but nonexclusive metabolic responses to FM, and these are functionally integrated into FM resistance mechanisms.

## Introduction

As an important leguminous crop, soybean is adapted to grow in a wide range of climatic conditions (Mutava et al., 2015); however, it is greatly affected by several abiotic and biotic stressors, including low temperature and high humidity (LTH). In Southwest China, soybeans are sown at the beginning of June and harvested at the end of October, during the autumn rainfall (Yang et al., 2014). During the autumn wet season, soybean crops that are in the field awaiting harvest are subjected to prolonged and continuous rainfall, which causes abnormally cold (temperature 13–21°C), humid (humidity 85–100%) weather (Deng et al., 2015). LTH stress during summer soybean seed development and maturity in the field also leads to pre-harvest field mold (FM). However, studies concerning LTH stress in soybean fruit, particularly its metabolic responses, are limited. Previous studies showed that black soybean exhibited better resistance to FM than common yellow soybeans, indicating that the seed coat is related to resistance to field mildew (Deng et al., 2015). However, there is still relatively little information concerning LTH stress responses of pre-harvest soybean. Field mold has become a significant obstacle to the enhancement of summer soybean production because the response mechanisms to mold damage remain unknown, as do the metabolic pathways that cause mildew-induced deterioration.

Metabonomic analysis is essential for understanding the ultimate responses of plant systems to genetic or environmental stresses (Glassbrook et al., 2000), and this method has emerged in recent years as a promising technology to identify metabolic networks in living plants (Fiehn, 2002). Untargeted metabonomic studies employing nuclear magnetic resonance (NMR) (Hagel et al., 2008) and chromatography-mass spectrometry (Schauer et al., 2006; Wen et al., 2014) have been used in various areas of biology, and these studies have utilized multiple detection and quantification strategies to collect large amounts of metabolic information. In particular, NMR analysis based on targeted profiling has made absolute qualitative and quantitative detection of metabolites more reliable (Suhre et al., 2011; Mousley et al., 2012), and a bioinformatics approach combined with this high-throughput metabonomics technique (Zhang et al., 2013a) makes it possible to use metabonomic strategies to reveal complex metabolic responses in the different organs of FM-damaged soybean fruit. The results of such studies will also aid in elucidating mechanisms of responses to LTH stress in soybean and other leguminous plants and assist in the breeding or engineering of soybean cultivars with resistance to LTH stress.

In the present study, the metabolic changes of mold-damaged soybean germplasms with different resistances to FM were investigated. The pods, seed coats and cotyledons were analyzed using NMR with multivariate data analysis, including principal component analysis (PCA), partial least-squares-discriminant analysis (PLS-DA), and analysis of the corresponding metabolic pathways. In addition, high-performance liquid chromatography (HPLC) were used to validate and predict the metabolic resistance mechanisms acquired from NMR profiles. Using these analyses, the complex specificity of metabolic reprogramming occurring in FM-damaged soybean fruit was also revealed, and potentially relevant structural genes and control elements were elucidated, ultimately facilitating the control of mildew in the field.

## Materials and methods

### Chemicals

Deuterium oxide (D_2_O, 99.9%) was purchased from Cambridge Isotope Laboratories Inc. (Miami, FL, USA). Anachro-certified DSS standard solution (ACDSS) was purchased from Anachro Technologies Inc. (Calgary, AB, Canada). The phosphate buffer solution (0.1 M K_2_HPO_4_/NaH_2_PO_4_, pH 7.29), formic acid and other chemicals used in this study were obtained from Sigma-Aldrich (St. Louis, MO, USA). HPLC-grade acetonitrile was obtained from Thermo Fisher Scientific Inc. (Waltham, MA, USA). All aqueous solutions were prepared using ultrapure water produced using a Milli-Q system (18.2 MX; Millipore, Bedford, MA, USA). All other chemicals used in NMR and UPLC-MS experiments were HPLC-grade.

### Materials and experimental design

The FM-susceptible variety “ND12,” which has a yellow seed coat and is a conventional cultivar in Southwestern China, and the highly resistant germplasm “C103,” which has a black seed coat and is grown in the Sichuan Province of China, were used in this study. The soybeans were grown in pots in the experimental field of the Sichuan Agricultural University at Ya'an in China (103°00′E, 30°08′N). Six seeds were sown per pot; these were thinned to three plants per pot 2 weeks after seeding. Half of the potted soybean plants were transferred from the field to a solar greenhouse approximately 5 days before growth stage R7 (beginning maturity), and other plants at the same stage of development were used as controls under normal conditions (20–30°C, humidity 60–70%). Plants in the greenhouse were exposed to a day/night temperature of 21/13°C and 85–100% humidity for 7 days during the remainder of the seed development and maturation period, according to Keigley et al. with some modifications (Keigley and Mullen, 1986). Three biological replicates of the above experiments were performed. Soybean fruits were harvested at the R8 stage (full maturity) of seed development, when mildew covered the LTH stress-damaged plants. The fruits from the middle portion of the treatment and control plants were collected, divided into three parts (pod, seed coat and cotyledon), and immediately frozen in liquid nitrogen, and the samples were stored in air-tight tubes at −80°C until further analysis.

### Metabolite sample preparation

Briefly, 50.00-mg freeze-dried samples were suspended in 1,000 μL of a 50%/50% methanol/water solution. Four cycles of a 4-second on/off cycling program were used for an in-solution ultrasonic extraction (Sonics VX-130, USA). Samples were centrifuged at 13,000 rpm for 15 min, and the supernatant was subsequently lyophilized and re-dissolved in 450 μL of water. This solution was transferred to a clean 2-mL centrifuge tube, and 50 μL of DSS (sodium 4,4-dimethyl-4-silapentane-1-sulfonate) standard D_2_O solution (Anachro, Canada) were added. Samples were mixed well and transferred to 5-mm NMR tubes (Norwell, USA).

### NMR spectra acquisition

Spectra were collected using a Bruker AV III 600 MHz spectrometer equipped with an inverse cryoprobe. The first increment of a 2D-^1^H, ^1^H-NOESY pulse sequence was utilized to acquire the ^1^H-NMR data and to suppress the solvent signal, and a MetNOESY pulse sequence was applied with a 100-ms mixing time and pre-saturation for 990 ms (~80 Hz gamma B1). Spectra were collected at 25°C with a total of 128 scans over a period of 15 min. The collected Free Induction Decay (FID) signal was automatically subjected to zero filling and Fourier transformation using a processing module in Chenomx NMR Suite 8.0 (Chenomx Inc., Edmonton, AB, Canada). The data were subsequently phased and baseline corrected using the Chenomx Processor. All spectra were referenced to the internal standard, DSS, and analyzed against the Chenomx Compound Library. From the 60 spectra, a total of 64 metabolites were identified and quantified. Data for the concentrations of all metabolites was exported to Microsoft Excel and normalized by weight across all parallel samples prior to use in subsequent multivariable analyses.

### HPLC-MS analysis

The concentrations of isoflavones and anthocyanins were analyzed using reversed phase high-pressure liquid chromatography (RP-HPLC) with electrospray ionization mass spectrometry (ESI-MS) detection. Extraction and chromatographic analysis procedures were based on previously published methods, with certain modifications (Zhang et al., 2011; Liu et al., 2016). Twelve common isoflavones and eight anthocyanins were quantified by external standardization using an Agilent 1260-series high performance liquid chromatography (HPLC) system equipped with a mass spectrometric detector (Agilent Quadrupole LC/MS 6120). The isoflavones and anthocyanins were identified by comparing the sample retention times and mass spectra with those of standard compounds, and absolute quantification was carried out via linear regression of corresponding standards.

### Data analysis

Chemometric analysis was performed using unsupervised PCA and supervised PLS-DA; PCA was employed to summarize the systematic alteration of samples using SIMCA-P 13.0 software (Umetrics, MKS Instruments Inc., Umea, Sweden). PLS-DA and pathway analysis were performed using MetaboAnalyst 3.0 (http://www.metaboanalyst.ca/) (Xia and Wishart, 2011). The original concentrations of all samples were normalized by summation, log transformation (generalized logarithm transformation or glog) and pareto scaling (mean-centering and division by the square root of the standard deviation of each variable). The permutation test, wherein each data point was randomly assigned to a class 1,000 times, was applied to evaluate the reliability of the model and prevent over-fitting (Westerhuis et al., 2010). Identification of important features was conducted based on variable influence on projection (VIP) scores. The metabolites for grouping, which had VIP > 1, were used to assess variables of significance for further scrutiny. ANOVA was performed using SPSS (version 20.0; SPSS, Chicago, IL, USA) to test the significance of differences between metabolite levels in the pod, seed coat and cotyledon of soybeans with and without FM. Differences were considered significant at *p* < 0.05.

## Results

### NMR spectroscopy and metabolite identification

The detected metabolites were identified based on comparisons with the spectra of standard compounds using the Chenomx NMR software suite (Figure S1) (Claesson et al., 2012). The metabolites identified in different soybean fruit organs, with their molecular masses, Kyoto Encyclopedia of Genes and Genomes (KEGG) compound codes, and other relevant information, are shown in Table S1, and typical ^1^H-NMR spectra of a 50% methanol soybean extract and main metabolite annotations are shown in Figure S2. In addition, the absolute concentrations of metabolites were obtained by integration of the isolated NMR signals with respect to the internal DSS standard. The concentrations of metabolites measured in organs of soybean fruits with different resistances to FM are shown in Table S2. Of these, 64 metabolites were identified as sugars, free amino acids, or organic acids, including 25 amino acids and their derivatives, 16 organic acids, 7 sugars and 16 other compounds (Table S2).

### Overview of the metabolic response

**Figure 1:**
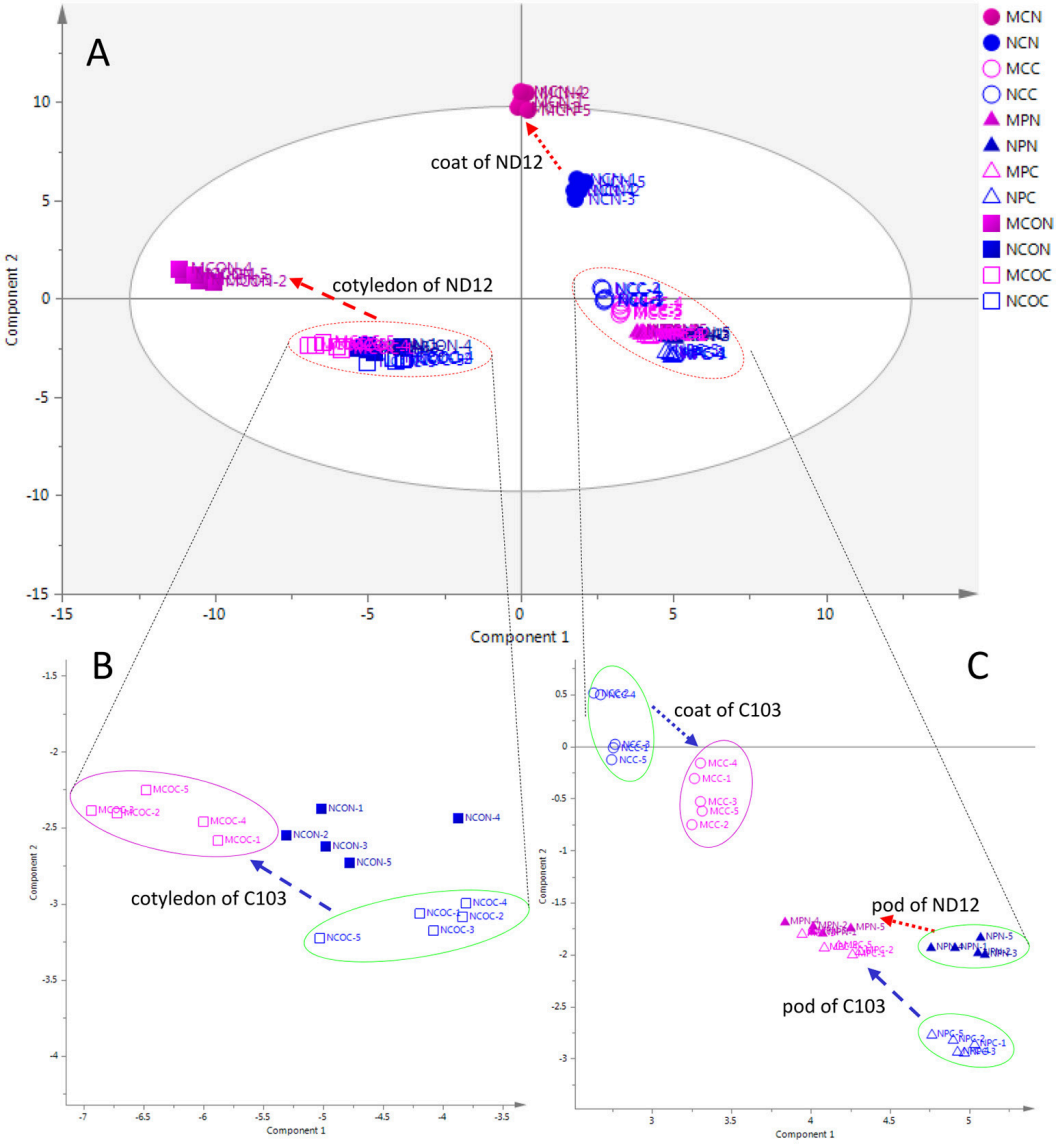
**PCA score plots of organ-specific differences in soybeans. (A)** Complete overview of PCA score plots. **(B)** Expanding PCA score plots of cotyledon. **(C)** Expanding PCA score plots of seed coat and pod. The ellipse represents the 95% confidence region for Hotelling's T2. Blue and red markers represent the control and mold-infected samples, respectively. Solid and hollow markers represent the susceptible variety ND12 and the mold-resistant variety C103, respectively. Circles, triangles, and boxes represent the seed coat, pod, and cotyledon, respectively. Red and blue arrows indicate the directions of changes from control samples to mold-infected samples of ND12 and C103, respectively. MCN, mold-infected seed coat of ND12; NCN, control seed coat of C103; MCC, mold-infected seed coat of C103; NCC, control seed coat of C103; MPN, mold-infected pod of ND12; NPN, control pod of ND12; MPC, mold-infected pod of C103; NPC, control pod of C103; MCON, mold-infected cotyledon of ND12; NCON, control cotyledon of ND12; MCOC, mold-infected cotyledon of C103; NCOC, control cotyledon of C103.

To investigate metabolic responses to FM in different organs of soybean fruits and to characterize the differences between mold-resistant and susceptible varieties, an unsupervised multivariate statistical analysis PCA was employed to obtain an overview of the data and to reveal the similarities and differences among all 12 groups of samples. In the PCA score plot (Figure 1), 60 collections (12^*^5) were separated into four groups. Figure 1A shows the PCA plot of all samples, showing discrimination between the cotyledon and other organs (seed coat and pod) in the first component and between the seed coat and pod in the second component. The metabolic profiles of the cotyledon and seed coat in the susceptible variety ND12 changed in response to FM. However, the metabolic profiles of the three organs of the resistant soybean C103 and the pods of ND12 were unchanged.

The expanding PCA score plot in the lower left of the first component (Figure 1B) indicated that the metabolic phenotypes of the control cotyledons were similar among these varieties with different mold resistances. In response to FM, the metabolic profiles of cotyledons in the susceptible variety ND12 showed a pronounced change. Although the metabolic profiles of cotyledons of the resistant variety C103 also changed slightly in response to FM, this alteration was not obvious. More details on the metabolic profiles of the seed coat and pod are shown in the expanding PCA score plot in the lower right of the first component (Figure 1C). This plot shows that the metabolic phenotypes of control seed coats in the studied soybean varieties were different. The metabolic profiles of the seed coat also changed in response to FM. Similar to the observations in cotyledons, changes in the metabolic profile of the seed coat of susceptible variety ND12 were more substantial than those in the resistant germplasm C103. In addition, the expanding PCA score plot of the seed coat indicated that the metabolic profiles of seed coats changed slightly in response to FM. Interestingly, the profiles of the mold-infected pods from the different varieties converged, although there was a distinct difference between their metabolic profiles and those of control pods (Figure 1C).

### Organ-specific metabolic analysis

**Figure 2:**
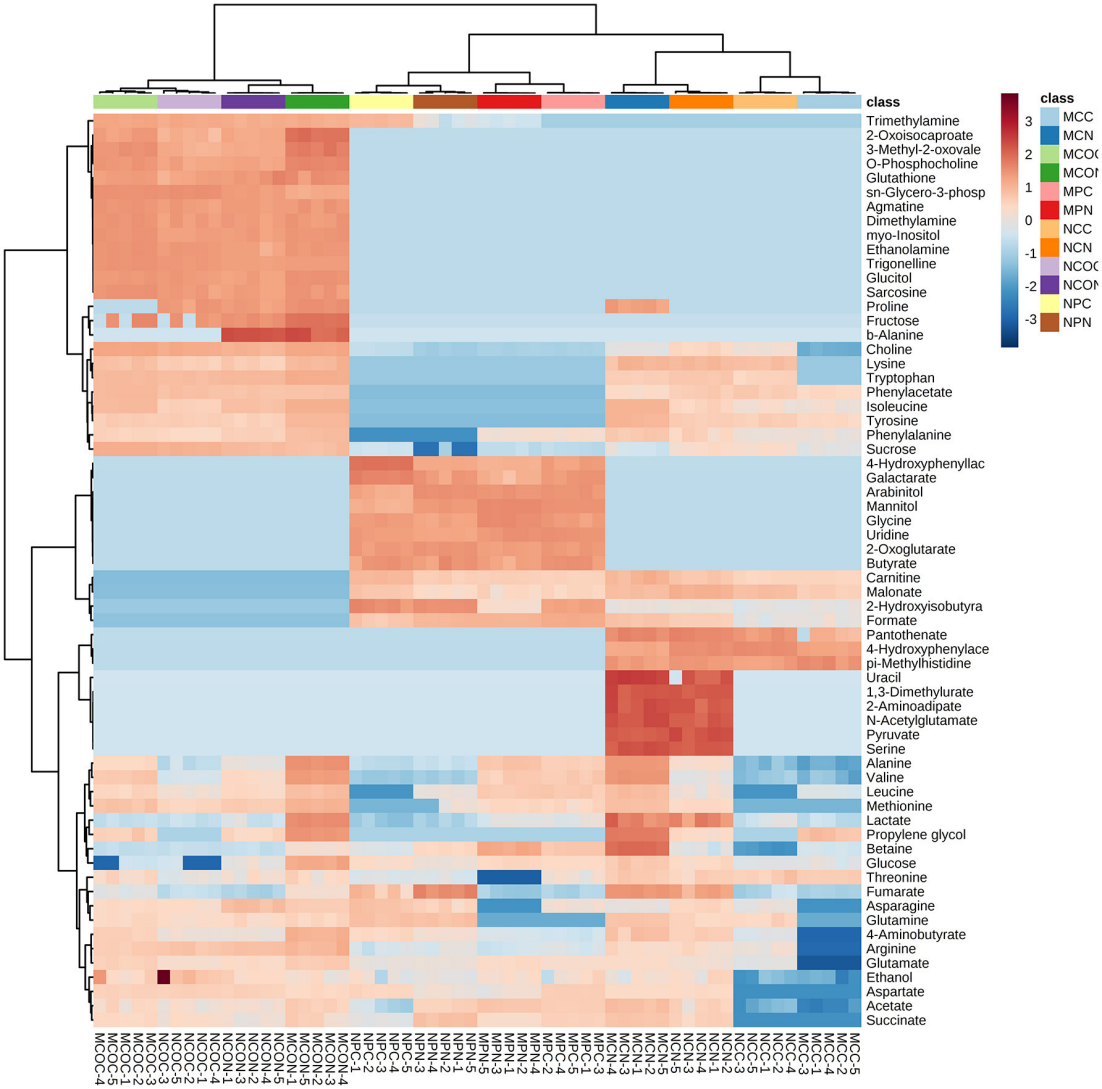
**Heat maps of the relative changes in metabolite abundance in different tissues of soybean fruits with and without FM**. Cell colors indicate normalized compound concentrations, with samples in rows and compounds in columns. The color scale at the right indicates the relative metabolite concentrations, with high concentrations in red and low concentrations in blue.

To select the most influential metabolites for discrimination between the treatment classes in the cotyledons, seed coats and pods of different soybean varieties, supervised PLS-DA models were applied. In the PLS-DA score plots of the cotyledon, seed coat, and pod (Figures S3–S5, respectively), the FM-damaged samples were clearly separated from their corresponding control samples based on the first principal component. As shown in Table S2, the contents of most of the metabolites in the cotyledons of both varieties increased in response to FM, but the functional importance of these metabolites in the discrimination of treatment classes was unclear. The calculated variable importance in the projection (VIP) scores of cotyledons of ND12 and C103 are presented in Figures S3E,F, respectively. The most influential metabolites in the discrimination of treatment classes were identified according to the VIP scores. Metabolites with a VIP score greater than 1 were considered important to the PLS–DA model. In the VIP-identified plot of ND12 cotyledons, 17 metabolites were selected as being most influential, including 5 organic acids, 3 sugars, and 7 amino acids; contents of these compounds increased exponentially in response to FM (Figure S3E, Table S2). A total of 17 metabolites, including 3 organic acids and 9 amino acids, were considered important to the PLS–DA model of C103 cotyledons (Figure S3F, Table S2); contents of almost all these metabolites increased exponentially in response to FM. In particular, as shown in the heat map generated from the normalized concentrations of all metabolites (Figure 2), the concentration of proline in C103 cotyledons (0.183–0.000 mg g^−^^1^) decreased significantly under LTH conditions (Figure 2, Table S2).

In contrast to observations in the cotyledons of both varieties, the contents of most of the metabolites measured in the seed coats of ND12 increased in response to FM, while decreases were observed in the seed coats of C103 (Figure 2, Table S2). In the VIP-identified plot of ND12 seed coats, 15 metabolites were selected as being the most influential in response to FM, including 2 organic acids with decreased contents and 10 amino acids with increased contents (Figure S4E, Table S2). In the seed coat of C103, however, 4-aminobutyrate and 6 amino acids, all of which had decreased contents, were selected as the most influential metabolites in response to FM (Figure S4E, Table S2). In particular, as shown in the heat map of Figure 2, the concentrations of proline (0.000–0.139 mg g^−^^1^), betaine (0.083–0.432 mg g^−^^1^), and valine (0.035–0.146 mg g^−^^1^) in ND12 increased significantly under LTH conditions. However, no significant change was detected in the seed coat of C103. Notably, these compounds are closely related to plant physiological resistance (Shen et al., 1997; Lu et al., 2014). However, contents of several other important metabolites decreased in the seed coat of C103 in response to FM, including choline (0.496–0.042 mg g^−^^1^), lysine (0.078–0.000 mg g^−^^1^), tryptophan (0.058–0.000 mg g^−^^1^), arginine (0.127–0.000 mg g^−^^1^), 4-aminobutyrate (0.030–0.000 mg g^−^^1^), and glutamate (0.177–0.000 mg g^−^^1^) (Figure 2, Table S2).

As shown in the heat map in Figure 2, the control and mold-infected profiles of cotyledons and seed coats in the two soybean varieties were similar. However, a substantial difference was observed between the profiles of control and mold-infected pods, and the concentrations of several metabolites were significantly higher in pods of ND12 than in C103, including pyruvate, uracil, 2-aminoadipate, N-acetylglutamate, serine, and 1,3-dimethylurate (dark red in Figure 2). Combinatorial analysis using the details in Table S2 revealed that under LTH conditions, more metabolites were detected at high concentrations in FM-damaged pods, particularly sugars (arabinitol, mannitol, and glucose) and ethanol. In the VIP-identified plot of pods of ND12 and C103, 9 and 15 metabolites (VIP score > 1), respectively, were selected as the most influential metabolites responding to FM (Figures S5E,F, Table S2). As shown in Table S2, most of the metabolites identified by PLS-DA as being important changed significantly, and most showed similar patterns of change in the **two** varieties, with the exceptions of succinate and arabinitol, which increased significantly in C103 and decreased in ND12 in response to FM under LTH conditions (Figure 2, Table S2).

### Analysis of the variation in metabolic pathways

**Figure 3:**
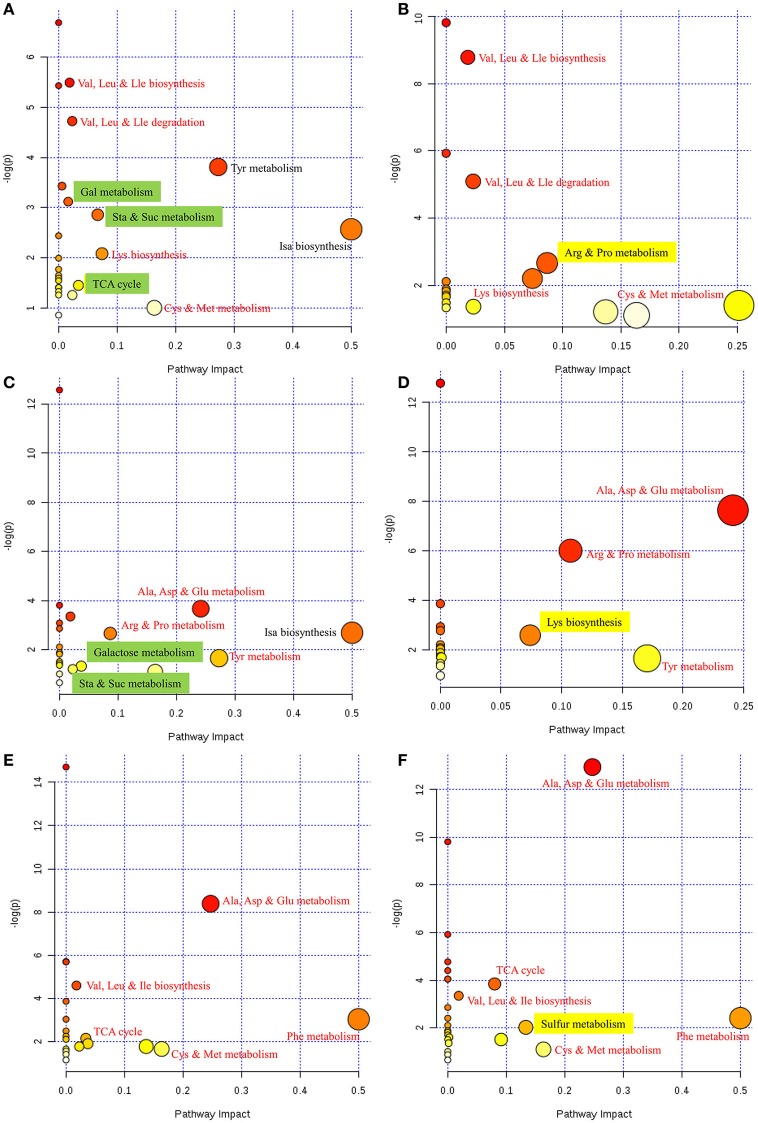
**Overview of pathway analyses indicating the metabolic pathways that are significantly affected in response to FM. (A)** Control vs. mold-infected cotyledon of ND12. **(B)** Control vs. mold-infected cotyledon of C103. **(C)** Control vs. mold-infected seed coat of ND12. **(D)** Control vs. mold-infected seed coat of C103. **(E)** Control vs. mold-infected pod of ND12. **(F)** Control vs. mold-infected pod of C103. Node size indicates the impact of each pathway (based on the impact of each identified metabolite in a given pathway), and the locations of the nodes indicate the significance of enrichment using -log(p). Metabolic pathways that are significantly impacted by FM appear in the upper right corner of each panel. Red text indicates that the same pathways responded to FM in the two varieties. Green highlighting indicates pathways that were affected only in the susceptible variety ND12. Yellow highlighting indicates pathways that were affected only in the resistant germplasm C103. Cys, cysteine; Met, methionine; Arg, arginine, Pro, proline, Gly, glycine, Ser, serine; Thr, threonine; Val, valine; Leu, leucine; Ile, isoleucine; Ala, alanine; Asp, aspartate; Glu, glutamate; Phe, phenylalanine; Tyr, tyrosine; Lys, lysine; Gal, galactose; Sta, starch; Suc, sucrose; Isa, isoquinoline alkaloid; Cys, cysteine; Met, methionine; TCA cycle, tricarboxylic acid cycle.

To further interpret this metabolic information in a biologically meaningful manner, enrichment and metabolite topology analyses were performed on the metabolites selected from PLS-DA. The KEGG pathway library of *Arabidopsis thaliana* was selected for comparison, and a specific hypergeometric test was used for the over-representation analysis. Relative betweenness centrality was selected as an importance measurement for topological analysis. An overview of the interactive visualization pathway analysis is shown in Figure 3; this was implemented to facilitate data exploration.

As shown in Figure 3A, metabolite differences in the cotyledons of control and FM samples of ND12 were enriched primarily in several metabolic pathways, including Val, Leu and Ile metabolism; Tyr metabolism; Gal metabolism; Sta and Suc metabolism; Isa biosynthesis; Lys biosynthesis; TCA cycle; and Cys and Met metabolism. In the cotyledons of C103 (Figure 3B), Val, Leu and Ile metabolism; Lys biosynthesis; Arg and Pro metabolism; and Cys and Met metabolism increased significantly in response to FM. Comparison of the responding metabolic pathways between these two varieties revealed that more metabolic pathways were significantly affected by FM in ND12 than in C103. For the most part, the same amino acid metabolic pathways, including Val, Leu, and Ile metabolism; Lys biosynthesis; Arg and Pro metabolism; and Cys and Met metabolism, were significantly affected in response to FM. However, the responses of several pathways of carbohydrate metabolism, including galactose metabolism, starch and sucrose metabolism, and the TCA cycle (highlighted in green in Figure 3A), differed in ND12 and C103 cotyledons (Figure 3B). In addition, arginine and proline metabolism responded differently in the cotyledons of these two varieties (highlighted in yellow in Figure 3B), which reflects the down-regulation of proline metabolism in the cotyledons of C103 in response to FM, whereas proline metabolism was up-regulated in ND12 cotyledons in response to FM (Figure 3A). This finding was also apparent in the heat map generated from the concentrations of all metabolites (Figure 2).

**Figure 4:**
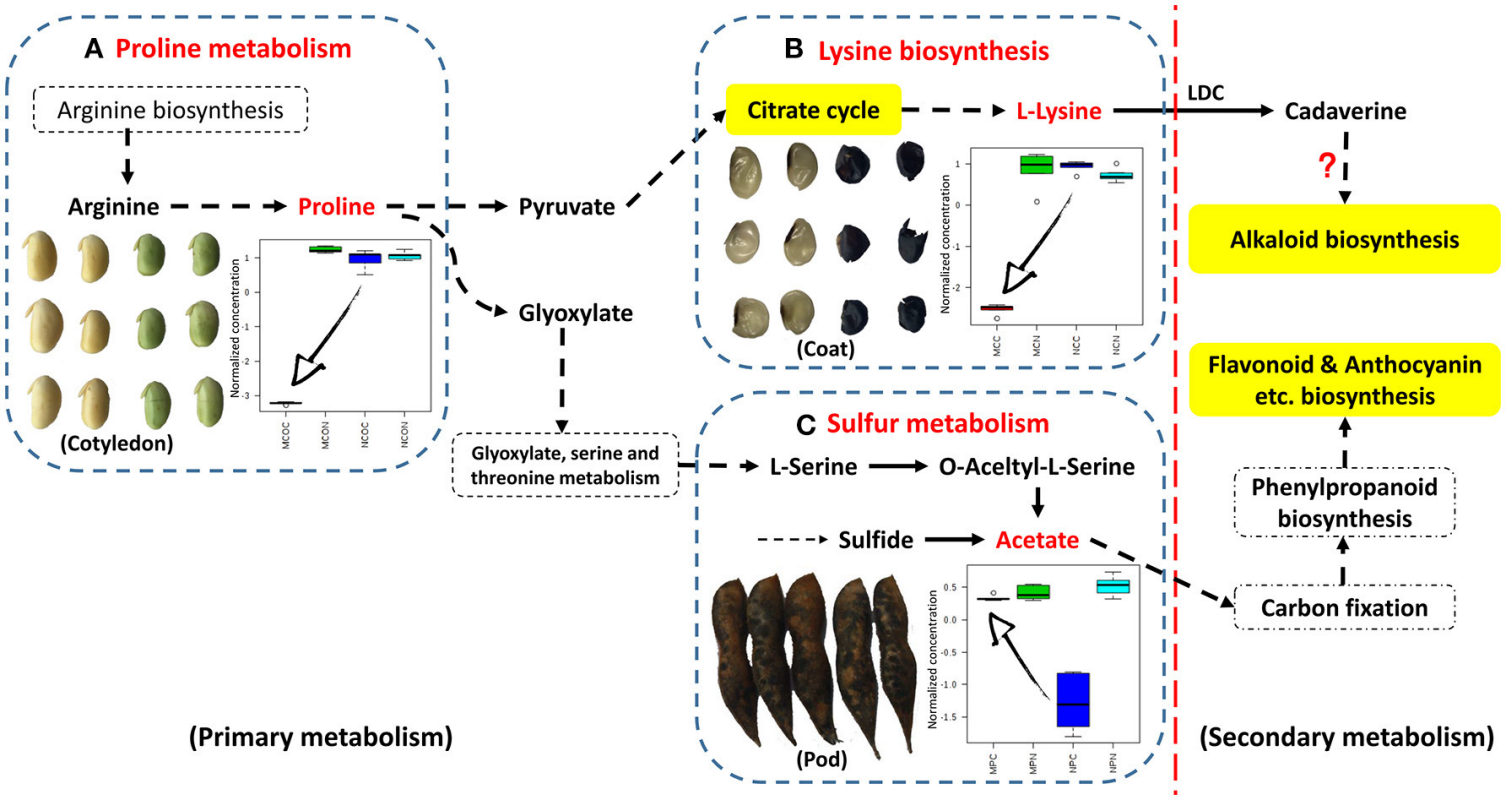
**Schematic showing the points at which FM affects metabolism in different tissues of soybean fruit investigated via targeted analysis**. This information was integrated and simplified from the metabolic pathways of *Glycine max* (soybean) in KEGG. **(A)** Relative changes in the normalized concentrations of proline in soybean cotyledons in response to FM; **(B)** lysine in soybean seed coats in response to FM; **(C)** acetate in soybean pods in response to FM (*n* = 5; error bars indicate standard deviation). LDC, lysine decarboxylase.

The metabolic pathways in the upper right corner were significantly impacted by FM (Figure 3). Pathway analyses of seed coats (Figures 3C,D) indicated the metabolic pathways that were significantly affected by FM. As was seen in cotyledons (Figures 3A,B), the responses of pathways involved in metabolism of carbohydrates, including galactose, starch and sucrose (highlighted in green in Figure 3C), differed in the seed coats of ND12 and C103. In addition, the same amino acid metabolic pathways, including Ala, Asp, and Glu metabolism; Tyr metabolism; and Arg and Pro metabolism, were observed to respond to FM in the seed coats of these two varieties. However, the degree of the response to FM degree of these metabolic pathways in the seed coat of C103 was stronger than in ND12 (red nodes in the upper right corner of Figure 3D). Additionally, Lys biosynthesis (highlighted in yellow in Figure 3D) in the seed coat of C103 may play an important role in resistance to FM, as reflected by the down-regulation of lysine in the seed coat of C103 in response to FM (Figure 4).

Further analysis of soybean pods indicated similar response patterns in these two varieties with differing resistance to FM. As shown in Figures 3E,F, the pathways with significantly affected responses were similar in ND12 and C103, including Ala, Asp, and Glu metabolism; Val, Leu, and Ile biosynthesis; Phe metabolism; Cys and Met metabolism; and the TCA cycle. In addition, pathways of sulfur metabolism were also significantly affected in the pod of C103 (highlighted in yellow in Figure 3F), indicating that sulfur metabolism in pods might play an important role in resistance to FM; this role is also reflected by the up-regulation of acetate in the pods of C103 in response to FM (Figure 4).

### Quantitative analysis of secondary metabolites

**Figure 5:**
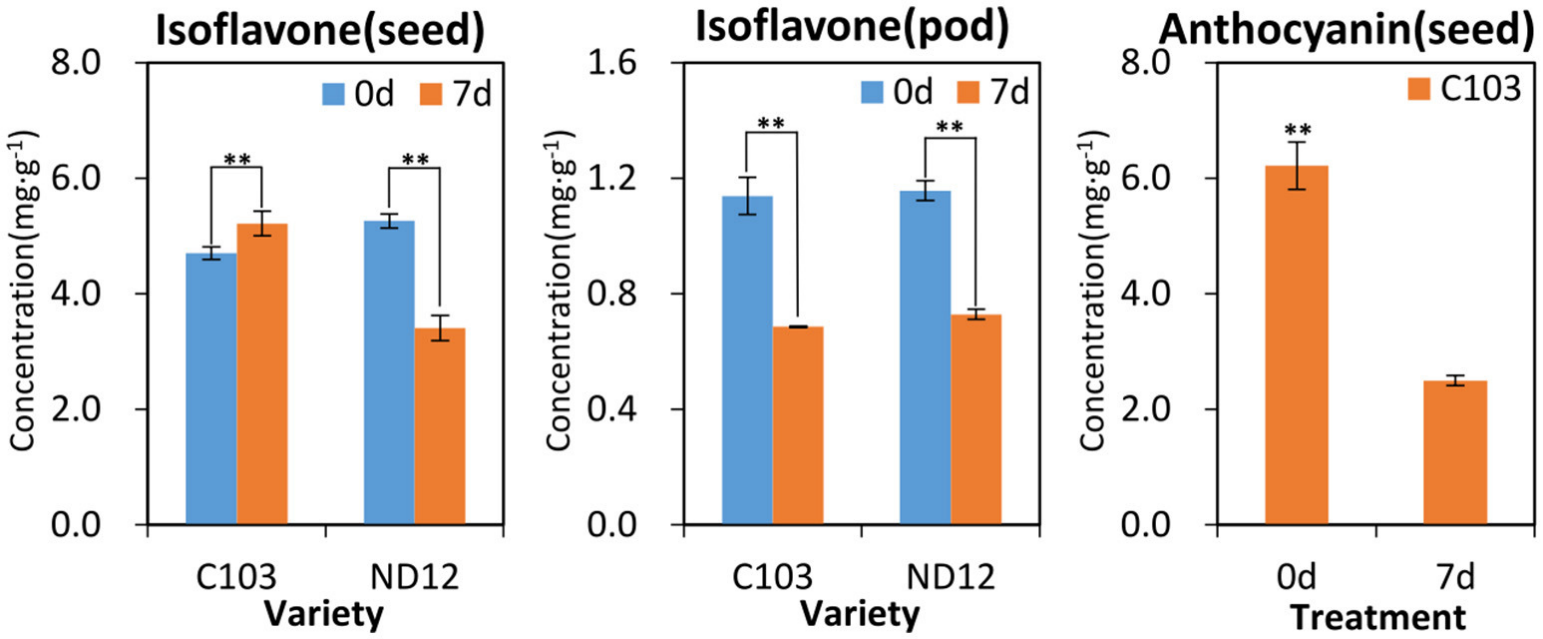
**Pre- and post-treatment contents of isoflavones and anthocyanins in soybean seeds and pods**. ^**^Significantly different (*p* < 0.01, Kruskall-Wallis test).

To further validate the results of the metabonomic analyses, the major isoflavones and anthocyanins were quantitatively analyzed by HPLC-MS (Figure S6). As shown in Figure 5, the isoflavones and anthocyanins in soybean seeds and pods decreased significantly after FM infection except in the seeds of the resistant variety C103, in which isoflavones and anthocyanins significantly increased after FM infection. Isoflavones, anthocyanins and their stress-induced derivatives (e.g., glyceollins) (Eromosele et al., 2012) are the products of the same phenylpropanoid pathways (Ferrer et al., 2008; Liu et al., 2015), and they are interconvertible *in planta* (Li et al., 2016). The decreased concentrations of isoflavones and anthocyanins implied an increase in other antibacterial compounds, particularly glyceollins (Kim et al., 2010). Therefore, metabonomic analyses indicated that phenylpropanoid metabolic pathways were indeed regulated in response to FM.

## Discussion

### The legume metabolome was strongly influenced by field mold

Soybean plants are often attacked by environmental microorganisms, particularly when subjected to low temperature and humidity stress. Among these microorganisms, the fungi *Aspergillus flavus, Aspergillus niger, Fusarium moniliforme*, and *Penicillium* are the major pathogens (described elsewhere). Biotic and abiotic stresses can result in soybean yield reduction and quality deterioration, such as decreased protein, fat and carbohydrate contents. Previous studies indicated that black soybean seeds exhibited less deterioration than common yellow soybean, and responses in different organs (cotyledon, seed coat, and pod) varied (described elsewhere). However, the mechanism of resistance was not clear. In the present study, it was hypothesized that organ-specific metabolic responses to FM may function in FM resistance, particularly in black soybean. In this study, targeted NMR profiling was used to compare metabonomic changes in pods, seed coats and cotyledons of different soybean varieties in response to FM under controlled LTH stress. The results were visualized using a PCA score plot, which indicated that all parts of the soybean fruits with different resistance were influenced to various degrees by FM. Overall, the metabolic profile of susceptible variety “ND12” showed greater responses to FM, particularly in the cotyledon and seed coat, whereas the resistant germplasm “C103” showed fewer changes in metabolism, particularly in the seed coat and pod. Previous studies have demonstrated that the secondary chemical constituents (high concentrations of anthocyanins) and physical structure (multiple layers of hard structures) of the black soybean seed coat determined resistance to various stresses such as drought, diseases and insects (Zhou et al., 2010; Wu et al., 2013; Zhang et al., 2013b).

In this study, additional resistance mechanisms on the level of primary metabolism were sought. The results revealed that the contents of several amino acids, amines, organic acids, sugars, and alcohols in soybean tissues were affected by FM infection, suggesting that amino acid, carbohydrate, and energy metabolism were affected to different extents by FM infection. For example, as previously described, several metabolic pathways involved in carbohydrate metabolism, including galactose metabolism, starch and sucrose metabolism, and the TCA cycle, exhibited different responses in the cotyledons and seed coats of ND12 and C103, suggesting that seed carbohydrate metabolism was easily activated in ND12, and more energy was consumed in ND12 than in C103 in response to FM under LTH stress. Carbohydrate metabolism plays an important role in pathogen resistance, such as through invertase-related tolerance and resistance to biotic and abiotic stresses (Balibrea Lara et al., 2004), which could be important for the specific modulation of host physiology in plant-microbe interactions and pathogen defense (Schäfer et al., 2015).

### As a signaling pathway, proline metabolism reflected the extent of damage to soybean cotyledons

Most of the nutrients in soybeans are stored in the cotyledons, and this determines the final quality of soybean seeds. Soybean cotyledons contain many amino acids, which are important nutrients that also function in plant resistance/tolerance physiology. Proline, in particular, accumulates in many plant species in response to environmental stress and functions both as the most beneficial compatible osmolyte in osmotic adjustment and in stabilization of protein structure and enzyme activity (Szabados and Savouré, 2010). Additional reports have indicated other functions in plant stress physiology, including maintenance of cellular homeostasis, scavenging for reactive oxygen species, energy supply, and functioning as a signaling molecule to influence metabolic networks during stress (Zhang et al., 2014). In the present study, changes in proline concentrations were found to differed markedly in different soybean varieties in response to FM infection. The proline contents of the cotyledons of the susceptible variety ND12 nearly doubled after infection, whereas its concentration decreased significantly in the resistant variety C103, suggesting that the cotyledons of susceptible ND12 were significantly damaged by FM, and their protective mechanisms were fully mobilized. In the resistant variety C103, however, the protection mechanism was not affected or may have been silenced under LTH stress. Proline accumulation can aid in tolerance of environmental stress, and proline can also function as a signaling molecule that reflects the extent of the damage suffered. The results of these experiments suggest that the decreased proline concentration in the cotyledon of C103 reflects high resistance to FM (Figure 4). The increase of proline in ND12, however, reflects its susceptibility to LTH stress. These results indicate that the soybean cotyledon, as the final nutrient sink, may be protected by other structures, such as the seed coat or pod.

### Lysine metabolism implies a new FM resistance mechanism involved in alkaloid biosynthesis in the soybean seed coat

Lysine is the major amino acid present in soybean seeds and is one of the eight essential amino acids that humans must obtain from food. Lysine decarboxylase (LDC) is the first key enzyme of the alkaloid biosynthetic pathways in some leguminous plants, and it plays an important role in metabolism (Bunsupa et al., 2012). After the decarboxylation of lysine, the secondary metabolic process of alkaloid biosynthesis is activated, and many alkaloids serve as antifungal factors (Hu et al., 2014). There are few reports concerning LDC and alkaloid biosynthesis in soybean. Although several LDC proteins were extracted and partially purified from soybeans decades ago (Kim et al., 1998; Ohe et al., 2009), no alkaloids were purified from soybean until recently, when several new indole-type alkaloids with a novel carbocyclic skeleton were isolated from green vegetable soybeans (Wang et al., 2016). This purification was the first known isolation of alkaloids from soybean, confirming alkaloid biosynthesis from lysine and indicating new biological significance for lysine biosynthesis in soybean. In the present study, lysine biosynthesis was highlighted in metabolic pathway analysis. The significant decrease in lysine in FM-infected seeds of resistant germplasm C103 indicated the activation of downstream secondary metabolic pathways, most likely alkaloid biosynthesis (Boschin and Resta, 2013). The biosynthesis and accumulation of alkaloids with high antifungal activity in the soybean seed coat may be another source of resistance to FM (Figure 4). Although this hypothesis has not been tested, the soybean seed coat plays an important role in FM resistance. Alkaloid biosynthesis from lysine in the soybean seed coat will be an excellent subject for future research.

### Sulfur metabolism in the soybean pod plays an important role in FM resistance through its involvement in phenylpropanoid biosynthesis

As the first line of defense, the legume pod plays a key role in the pathogen resistance. A previous study indicated that mildew on soybeans pods can mitigate the damage to the seed arising from FM during harvest (described elsewhere), but the mechanism was not clear. In the present study, as was the case for lysine biosynthesis in the seed coat, sulfur metabolism was highlighted in pathway analysis of soybean pods. Few studies have been conducted on plant sulfur metabolism although this element likely plays essential roles in resistance to biotic and abiotic stressors, including drought, cold, disease and insect attack, that ultimately affecting crop yield and grain quality (Yu et al., 2007), and sulfur metabolism has been found to act against fungal pathogens in plants (Bloem et al., 2007). Based on the results of previous studies, it is proposed that some resistance factors in soybean pods involve sulfur metabolism. The KEGG pathway analysis in the present study showed that acetate is located in the last step of sulfur metabolism and the first step of the carbon fixation pathway (Figure 4). The significant increase in acetate contents observed in FM-damaged fruits of resistant germplasm C103 indicated that sufficient carbon was supplied for downstream secondary metabolism, including production of isoflavones, anthocyanins and other phenolics, which also functions as protective agents against various stresses. The observed increase in sulfur metabolism enhanced sulfur utilization, leading to plant resistance, and stimulated downstream secondary metabolism for increased resistance to FM (Figure 4). The chromatographic analysis of isoflavones and anthocyanins confirmed this prediction (Figure 5). In summary, it is proposed that sulfur metabolism in soybean pods may play an important role in FM resistance, and the mechanism of this resistance is closely associated with other secondary metabolic pathways, such as the phenylpropanoid pathway.

## Conclusions

The black soybean variety studied here showed better resistance to FM infection than the yellow variety. Possible resistance mechanisms include both the physical structure and chemical constituents of the fruits, i.e., multiple hard layers and phenolics in the dark-colored seed coat, which could effectively prevent external damage from fungal infection following LTH stress in the field. Comparative organ-specific NMR metabolite analyses of black and yellow soybeans revealed additional resistance mechanisms at both the primary and secondary levels. In cotyledons, proline acts as a signal molecule, and proline metabolism determined the degree of damage to cotyledons. The seed coat and pod act as the primary physical and chemical barriers and function primarily in external mitigation, particularly via lysine biosynthesis and sulfur metabolism. Although additional studies are needed to confirm their functions, it is clear that the metabolic responses of different tissues determine the FM resistance of soybean fruits (Supplementary Presentation 1). The findings presented here will be useful in understanding the distinct but nonexclusive defense mechanisms in different tissues of soybean fruits and identification of the metabolic pathways associated with responses to and complex interactions against FM.

## Author contributions

Study idea and design: JL and WY. Field and lab work: JD, CY, and JZ. Data analysis: FY and QZ. Paper concept and writing: JL and JD. All authors discussed the results and provided comments at all stages of the manuscript.

### Conflict of interest statement

The authors declare that the research was conducted in the absence of any commercial or financial relationships that could be construed as a potential conflict of interest.
