# Supplementary material for: Organ-Specific Differential NMR-Based Metabonomic Analysis of Soybean [Glycine max (L.) Merr.] Fruit Reveals the Metabolic Shifts and Potential Protection Mechanisms Involved in Field Mold Infection
Source: Front Plant Sci. 2017 Apr 25;8:508. doi: 10.3389/fpls.2017.00508 (PMC5404178; doi:10.3389/fpls.2017.00508)
Supplement: Supplementary file 1 [file Table1.PDF]

Table S1. All metabolites identified in soybean fruit, with their molecular masses, KEGG compound codes, and other relevant information.

	CAS Registry	ChEBI ID	Compound ID	Formula	HMDB Accession Number	KEGG Compound ID	PubChem Compound	Weight
1,3-Dimethylurate	944-73-0		346	C7H8N4O3	HMDB01857		70346	196.1634
2-Aminoadipate	1118-90-7	CHEBI:17082	112	C6H11NO4	HMDB00510	C00956	92136	161.1558
2-Hydroxyisobutyrate	594-61-6		69	C4H8O3	HMDB00729		11671	104.1045
2-Oxoglutarate	328-50-7	CHEBI:30915	181	C5H6O5	HMDB00208	C00026	51	146.0981
2-Oxoisocaproate	816-66-0	CHEBI:17865	153	C6H10O3	HMDB00695	C00233	70	130.1418
3-Methyl-2-oxovalerate	1460-34-0		182	C6H10O3	HMDB00491	C03465	47	130.1418
4-Aminobutyrate	1956-12-2	CHEBI:16865	101	C4H9NO2	HMDB00112	C00334	119	103.1198
4-Hydroxyphenylacetate	156-38-7	CHEBI:18101	7	C8H8O3	HMDB00020	C00642	127	152.1473
4-Hydroxyphenyllactate	306-23-0	CHEBI:17385	96	C9H10O4	HMDB00755	C03672	9378	182.1733
Acetate	64-19-7	CHEBI:15366	9	C2H4O2	HMDB00042	C00033	176	60.052
Agmatine	306-60-5	CHEBI:17431	1733	C5H14N4	HMDB01432	C00179	199	130.1915
Alanine	56-41-7	CHEBI:16977	232	C3H7NO2	HMDB00161	C00041	5950	89.0932
Arabinitol	488-82-4	CHEBI:18333	48	C5H12O5	HMDB00568	C01904	94154	152.1458
Arginine	74-79-3	CHEBI:16467	240	C6H14N4O2	HMDB00517	C00062	6322	174.201
Asparagine	70-47-3	CHEBI:17196	231	C4H8N2O3	HMDB00168	C00152	6267	132.1179
Aspartate	56-84-8	CHEBI:17053	234	C4H7NO4	HMDB00191	C00049	5960	133.1027
Betaine	107-43-7	CHEBI:17750	15	C5H11NO2	HMDB00043	C00719	248	117.1463
Butyrate	107-92-6	CHEBI:30772	128	C4H8O2	HMDB00039	C00246	264	88.1051
Carnitine	541-15-1	CHEBI:16347	21	C7H15NO3	HMDB00062	C00318	10917	161.1989
Choline	62-49-7	CHEBI:15354	302	C5H14NO	HMDB00097	C00114	305	104.1708
Citrate	77-92-9	CHEBI:30769	22	C6H8O7	HMDB00094	C00158	311	192.1235
Dimethylamine	124-40-3	CHEBI:17170	27	C2H7N	HMDB00087	C00543	674	45.0837
Ethanol	64-17-5	CHEBI:16236	205	C2H6O	HMDB00108	C00469	702	46.0684
Ethanolamine	141-43-5	CHEBI:16000	97	C2H7NO	HMDB00149	C00189	700	61.0831
Formate	64-18-6	CHEBI:30751	32	CH2O2	HMDB00142	C00058	284	46.0254
Fructose	57-48-7	CHEBI:15824	86	C6H12O6	HMDB00660	C00095	439163	180.1559
Fumarate	110-17-8	CHEBI:18012	100	C4H4O4	HMDB00134	C00122	723	116.0722
Galactarate	526-99-8	CHEBI:30852	396	C6H10O8	HMDB00639	C00879	607	210.1388
Glucitol	50-70-4	CHEBI:17924	259	C6H14O6	HMDB00247	C00794	5780	182.1718
Glucose	50-99-7	CHEBI:17634	183	C6H12O6	HMDB00122	C00031	5793	180.1559
Glutamate	56-86-0	CHEBI:16015	229	C5H9NO4	HMDB00148	C00025	33032	147.1293
Glutamine	56-85-9	CHEBI:18050	226	C5H10N2O3	HMDB00641	C00064	5961	146.1445
Glutathione	70-18-8	CHEBI:16856	493	C10H17N3O6S	HMDB00125	C00051	124886	307.3235
Glycine	56-40-6	CHEBI:15428	242	C2H5NO2	HMDB00123	C00037	750	75.0666
Isoleucine	73-32-5	CHEBI:17191	225	C6H13NO2	HMDB00172	C00407	6306	131.1729
Lactate	79-33-4	CHEBI:422	42	C3H6O3	HMDB00190	C00186	107689	90.0779
Leucine	61-90-5	CHEBI:15603	235	C6H13NO2	HMDB00687	C00123	6106	131.1729
Lysine	56-87-1	CHEBI:18019	291	C6H14N2O2	HMDB00182	C00047	5962	146.1876
Malate	6915-15-7	CHEBI:6650	94	C4H6O5	HMDB00156	C00711	525	134.0874
Malonate	141-82-2	CHEBI:30794	121	C3H4O4	HMDB00691	C00383	867	104.0615
Mannitol	69-65-8	CHEBI:16899	201	C6H14O6	HMDB00765	C00392	6251	182.1718
Methionine	63-68-3	CHEBI:16643	213	C5H11NO2S	HMDB00696	C00073	6137	149.2113
N-Acetylglutamate	1188-37-0	CHEBI:17533	424	C7H11NO5	HMDB01138	C00624	70914	189.1659
O-Phosphocholine	107-73-3	CHEBI:18132	321	C5H15NO4P	HMDB00284	C00588	1014	184.1507
Pantothenate	79-83-4	CHEBI:18701	256	C9H17NO5	HMDB00210	C00864	6613	219.235
Phenylacetate	103-82-2	CHEBI:30745	50	C8H8O2	HMDB00209	C07086	999	136.1479
Phenylalanine	63-91-2	CHEBI:17295	227	C9H11NO2	HMDB00159	C00079	6140	165.1891
Proline	147-85-3	CHEBI:17203	230	C5H9NO2	HMDB00162	C00148	145742	115.1305
Propylene glycol	57-55-6	CHEBI:16997	262	C3H8O2	HMDB01881	C00583	1030	76.0944
Pyruvate	127-17-3	CHEBI:32816	133	C3H4O3	HMDB00243	C00022	1060	88.0621
Sarcosine	107-97-1	CHEBI:15611	57	C3H7NO2	HMDB00271	C00213	1088	89.0932
Serine	56-45-1	CHEBI:17115	216	C3H7NO3	HMDB00187	C00065	5951	105.0926
Succinate	110-15-6	CHEBI:15741	60	C4H6O4	HMDB00254	C00042	1110	118.088
Sucrose	57-50-1	CHEBI:17992	193	C12H22O11	HMDB00258	C00089	5988	342.2965
Threonine	72-19-5	CHEBI:16857	219	C4H9NO3	HMDB00167	C00188	6288	119.1192
Trigonelline	535-83-1	CHEBI:18123	334	C7H7NO2	HMDB00875	C01004	5571	137.136
Trimethylamine	75-50-3	CHEBI:18139	62	C3H9N	HMDB00906	C00565	1146	59.1103
Tryptophan	73-22-3	CHEBI:16828	241	C11H12N2O2	HMDB00929	C00078	6305	204.2252
Tyrosine	60-18-4	CHEBI:17895	243	C9H11NO3	HMDB00158	C00082	6057	181.1885
Uracil	66-22-8	CHEBI:17568	139	C4H4N2O2	HMDB00300	C00106	1174	112.0868
Uridine	58-96-8	CHEBI:16704	162	C9H12N2O6	HMDB00296	C00299	6029	244.2014
Valine	72-18-4	CHEBI:16414	215	C5H11NO2	HMDB00883	C00183	6287	117.1463
myo-Inositol	87-89-8	CHEBI:17268	223	C6H12O6	HMDB00211	C00137	892	180.1559
sn-Glycero-3-phosphocholine	28319-77-9	CHEBI:16870	333	C8H21NO6P	HMDB00086	C00670	439285	258.2292
β-Alanine	107-95-9	CHEBI:16958	67	C3H7NO2	HMDB00056	C00099	239	89.0932
π-Methylhistidine	368-16-1		76	C7H11N3O2	HMDB00479	C01152	64969	169.1811
