# Supplementary material for: Organ-Specific Differential NMR-Based Metabonomic Analysis of Soybean [Glycine max (L.) Merr.] Fruit Reveals the Metabolic Shifts and Potential Protection Mechanisms Involved in Field Mold Infection
Source: Front Plant Sci. 2017 Apr 25;8:508. doi: 10.3389/fpls.2017.00508 (PMC5404178; doi:10.3389/fpls.2017.00508)
Supplement: Supplementary file 2 [file Table2.pdf]

Table S2. Contents of 64 metabolites identified in soybean [*Glycine max* (L.) Merr.] fruit.

Metabolite	Pod (mg/g)				Seed coat (mg/g)				Cotyledon (mg/g)			
	ND12		C103		ND12		C103		ND12		C103	
	MCN	NCN	MCC	NCC	MPN	NPN	MPC	NPC	MCON	NCON	MCOC	NCOC
1,3-Dimethylurate	0.0440±0.0065	0.0369±0.0021	nd	nd	nd	nd	nd	nd	nd	nd	nd	nd
2-Aminoadipate	0.1204±0.0195	0.0872±0.0124	nd	nd	nd	nd	nd	nd	nd	nd	nd	nd
2-Hydroxyisobutyrate	0.0031±0.0002	0.0034±0.0001	0.0025±0.0002	0.0020±0.0002	0.0068±0.0004	0.1796±0.0063	0.0978±0.0023	0.2015±0.0283	nd	nd	nd	nd
2-Oxoglutarate	nd	nd	nd	nd	0.0499±0.0019	0.0743±0.0024	0.0644±0.0043	0.0616±0.0039	nd	nd	nd	nd
2-Oxoisocaproate	nd	nd	nd	nd	nd	nd	nd	nd	0.0285±0.0015	0.0067±0.0004	0.0092±0.0007	0.0047±0.0005
3-Methyl-2-oxovalerate	nd	nd	nd	nd	nd	nd	nd	nd	0.0114±0.0007	0.0039±0.0000	0.0069±0.0004	0.0034±0.0003
4-Aminobutyrate	0.2296±0.0251	0.1533±0.0068	nd	0.0298±0.0018	0.0254±0.0011	0.0691±0.0022	0.0193±0.0012	0.1123±0.0061	0.3774±0.0228	0.0527±0.0025	0.1449±0.0046	0.0570±0.0045
4-Hydroxyphenylacetate	0.0481±0.0026	0.1495±0.0049	0.0656±0.0028	0.1660±0.0051	nd	nd	nd	nd	nd	nd	nd	nd
4-Hydroxyphenyllactate	nd	nd	nd	nd	0.0135±0.0003	0.0180±0.0002	0.0264±0.0022	0.1153±0.0035	nd	nd	nd	nd
Acetate	0.3738±0.0079	0.2643±0.0225	0.0212±0.0023	0.0392±0.0050	0.3416±0.0044	0.2873±0.0077	0.3046±0.0028	0.0964±0.0153	0.2942±0.0086	0.2117±0.0068	0.2447±0.0183	0.2004±0.0132
Agmatine	nd	nd	nd	nd	nd	nd	nd	nd	0.0771±0.0065	0.0693±0.0033	0.0872±0.0025	0.0646±0.0065
Alanine	0.3135±0.0011	0.1150±0.0024	0.0176±0.0012	0.0242±0.0020	0.1738±0.0035	0.0514±0.0011	0.1470±0.0016	0.0454±0.0017	0.3375±0.0038	0.0830±0.0022	0.1281±0.0013	0.0479±0.0038
Arabinitol	nd	nd	nd	nd	0.7238±0.0530	0.9354±0.0351	0.7480±0.0499	0.2338±0.0448	nd	nd	nd	nd
Arginine	0.2467±0.0282	0.2268±0.0306	nd	0.1269±0.0155	0.0388±0.0045	0.0752±0.0113	0.0569±0.0063	0.0501±0.0071	0.9998±0.1195	0.6867±0.0177	0.4553±0.0260	0.5780±0.0299
Asparagine	0.0489±0.0028	0.2084±0.0134	nd	0.0484±0.0057	nd	0.2316±0.0342	0.0837±0.0090	0.4970±0.0357	0.3163±0.0096	0.6215±0.0464	0.1746±0.0095	0.1472±0.0137
Aspartate	0.1727±0.0004	0.1818±0.0076	nd	nd	0.0787±0.0123	0.0801±0.0086	0.3267±0.0078	0.2298±0.0088	0.1968±0.0080	0.2788±0.0124	0.2748±0.0122	0.2883±0.0076
Betaine	0.4317±0.0082	0.0834±0.0057	0.0712±0.0016	0.0198±0.0008	0.2381±0.0053	0.1344±0.0014	0.1669±0.0025	0.0793±0.0021	0.1139±0.0019	0.0606±0.0014	0.0584±0.0009	0.0598±0.0011
Butyrate	nd	nd	nd	nd	0.0060±0.0006	0.0086±0.0012	0.0091±0.0007	0.0082±0.0009	nd	nd	nd	nd
Carnitine	0.0921±0.0113	0.0430±0.0022	0.0230±0.0004	0.0225±0.0007	0.0294±0.0009	0.0309±0.0018	0.0253±0.0007	0.0794±0.0027	nd	nd	nd	nd
Choline	0.3386±0.0055	0.7276±0.0242	0.0424±0.0016	0.4956±0.0169	0.1064±0.0017	0.0928±0.0019	0.1082±0.0014	0.1656±0.0034	1.9518±0.0269	1.5384±0.0372	2.0281±0.0261	1.8113±0.0348
Dimethylamine	nd	nd	nd	nd	nd	nd	nd	nd	0.0111±0.0007	0.0101±0.0005	0.0124±0.0004	0.0099±0.0009
Ethanol	0.0101±0.0005	0.0112±0.0006	0.0038±0.0003	0.0042±0.0003	0.0111±0.0002	0.0091±0.0001	0.0103±0.0010	0.0092±0.0009	0.0105±0.0005	0.0118±0.0003	0.0132±0.0015	0.0238±0.0097
Ethanolamine	nd	nd	nd	nd	nd	nd	nd	nd	0.1532±0.0051	0.0956±0.0133	0.1842±0.0064	0.1477±0.0070
Formate	0.0175±0.0006	0.0113±0.0002	0.0029±0.0005	0.0022±0.0002	0.0297±0.0004	0.0274±0.0010	0.0411±0.0006	0.0150±0.0011	nd	nd	nd	nd
Fructose	nd	nd	nd	nd	nd	nd	nd	nd	3.3009±0.1162	0.7110±0.0799	0.6031±0.2556	0.3315±0.1446
Fumarate	0.0816±0.0009	0.0679±0.0047	0.0095±0.0002	0.0115±0.0015	0.0082±0.0007	0.0937±0.0040	0.0103±0.0006	0.0447±0.0053	0.0277±0.0004	0.0101±0.0005	0.0198±0.0007	0.0132±0.0006
Galactarate	nd	nd	nd	nd	0.0190±0.0028	0.0520±0.0077	0.0563±0.0046	0.1130±0.0061	nd	nd	nd	nd
Glucitol	nd	nd	nd	nd	nd	nd	nd	nd	3.6749±0.2469	1.4330±0.1727	2.4754±0.1901	2.2067±0.1858
Glucose	1.2752±0.0969	0.6168±0.0496	0.1526±0.0233	0.1308±0.0059	0.7110±0.0281	0.2860±0.0135	0.6041±0.0023	0.5228±0.0283	4.0723±0.3362	0.2134±0.0367	0.0393±0.0161	0.0320±0.0197
Glutamate	0.5587±0.0052	0.3513±0.0148	nd	0.1771±0.0115	0.6009±0.0283	0.2640±0.0066	0.4830±0.0069	0.2287±0.0141	1.0657±0.0391	0.5829±0.0237	0.8411±0.0374	0.4435±0.0140
Glutamine	0.1986±0.0041	0.0836±0.0075	nd	0.0870±0.0115	nd	0.2152±0.0143	nd	0.2350±0.0127	0.0859±0.0077	0.0652±0.0090	0.0759±0.0067	0.0587±0.0046
Glutathione	nd	nd	nd	nd	nd	nd	nd	nd	0.0841±0.0107	0.0786±0.0173	0.0528±0.0025	0.0390±0.0060
Glycine	nd	nd	nd	nd	0.0638±0.0025	0.0263±0.0017	0.0366±0.0031	0.0372±0.0014	nd	nd	nd	nd
Isoleucine	0.0913±0.0009	0.0220±0.0018	0.0084±0.0011	0.0100±0.0013	nd	nd	nd	nd	0.1101±0.0008	0.0524±0.0015	0.0679±0.0011	0.0250±0.0009
Lactate	0.5541±0.0693	0.4793±0.0480	0.0624±0.0046	0.0855±0.0088	0.0997±0.0050	0.0490±0.0033	0.1066±0.0066	0.0378±0.0032	0.4763±0.0049	0.0812±0.0081	0.0657±0.0019	0.0657±0.0025
Leucine	0.1013±0.0042	0.0189±0.0026	0.0078±0.0005	nd	0.0642±0.0024	0.0150±0.0008	0.0654±0.0046	nd	0.0952±0.0044	0.0357±0.0021	0.0425±0.0029	0.0197±0.0015
Lysine	0.1254±0.0215	0.1096±0.0090	nd	0.0783±0.0055	nd	nd	nd	nd	0.1184±0.0127	0.0409±0.0062	0.0774±0.0072	0.0380±0.0030
Malonate	0.0234±0.0005	0.0380±0.0015	0.0129±0.0008	0.0230±0.0008	0.0089±0.0015	0.0076±0.0005	0.0095±0.0007	0.0176±0.0010	nd	nd	nd	nd
Mannitol	nd	nd	nd	nd	1.6100±0.0225	0.7372±0.0289	1.0283±0.0208	0.1854±0.0202	nd	nd	nd	nd
Methionine	0.0333±0.0003	0.0132±0.0005	nd	nd	0.0138±0.0012	0.0035±0.0016	0.0134±0.0015	nd	0.0531±0.0012	0.0214±0.0010	0.0292±0.0016	0.0149±0.0006
N-Acetylglutamate	0.0110±0.0008	0.0085±0.0006	nd	nd	nd	nd	nd	nd	nd	nd	nd	nd
O-Phosphocholine	nd	nd	nd	nd	nd	nd	nd	nd	0.0712±0.0017	0.0268±0.0016	0.0432±0.0016	0.0247±0.0023
Pantothenate	0.0159±0.0012	0.0169±0.0007	0.0037±0.0011	0.0111±0.0010	nd	nd	nd	nd	nd	nd	nd	nd
Phenylacetate	0.0479±0.0042	0.1248±0.0122	0.0629±0.0045	0.1380±0.0128	nd	nd	nd	nd	0.2113±0.0057	0.1933±0.0091	0.3415±0.0119	0.2306±0.0127
Phenylalanine	0.1015±0.0043	0.0626±0.0093	0.0227±0.0024	0.0244±0.0006	0.0313±0.0016	nd	0.0373±0.0021	nd	0.1508±0.0053	0.0782±0.0019	0.0668±0.0027	0.0527±0.0022
Proline	0.1387±0.0196	nd	nd	nd	nd	nd	nd	nd	0.2624±0.0119	0.1514±0.0166	nd	0.1832±0.0237
Propylene glycol	0.0262±0.0005	0.0017±0.0001	0.0039±0.0003	nd	nd	nd	nd	nd	0.0135±0.0004	0.0019±0.0002	0.0026±0.0003	nd
Pyruvate	0.0212±0.0006	0.0289±0.0032	nd	nd	nd	nd	nd	nd	nd	nd	nd	nd
Sarcosine	nd	nd	nd	nd	nd	nd	nd	nd	0.0056±0.0004	0.0046±0.0002	0.0061±0.0002	0.0051±0.0003
Serine	0.1007±0.0107	0.0773±0.0044	nd	nd	nd	nd	nd	nd	nd	nd	nd	nd
Succinate	0.1128±0.0030	0.0695±0.0060	nd	nd	0.0410±0.0012	0.1333±0.0072	0.0718±0.0014	0.0140±0.0010	0.0609±0.0136	0.0253±0.0034	0.0540±0.0025	0.0417±0.0048
Sucrose	0.3545±0.1391	2.5004±0.3085	0.5844±0.0654	2.2518±0.2354	0.1048±0.0132	0.0074±0.0074	0.0899±0.0106	0.1836±0.0079	12.0450±0.8650	14.6331±0.7722	27.5691±0.3469	23.2371±0.9444
Threonine	0.0990±0.0056	0.0807±0.0082	0.0915±0.0016	0.0931±0.0122	nd	0.0190±0.0019	0.0394±0.0043	0.0201±0.0012	0.0618±0.0101	0.0436±0.0068	0.0607±0.0072	0.0246±0.0027
Trigonelline	nd	nd	nd	nd	nd	nd	nd	nd	0.3379±0.0047	0.2906±0.0046	0.4943±0.0061	0.4052±0.0094
Trimethylamine	nd	nd	nd	nd	0.0004±0.0000	0.0006±0.0001	nd	0.0050±0.0001	0.0068±0.0002	0.0069±0.0003	0.0084±0.0002	0.0075±0.0003
Tryptophan	0.0789±0.0032	0.1225±0.0049	nd	0.0584±0.0054	nd	nd	nd	nd	0.5224±0.0135	0.2864±0.0072	0.2563±0.0075	0.2151±0.0097
Tyrosine	0.1596±0.0048	0.0519±0.0020	0.0313±0.0021	0.0500±0.0038	nd	nd	nd	nd	0.1464±0.0024	0.0422±0.0022	0.0546±0.0032	0.0521±0.0019
Uracil	0.0125±0.0006	0.0057±0.0016	nd	nd	nd	nd	nd	nd	nd	nd	nd	nd
Uridine	nd	nd	nd	nd	0.0422±0.0037	0.0224±0.0007	0.0426±0.0021	0.0301±0.0014	nd	nd	nd	nd
Valine	0.1463±0.0016	0.0352±0.0019	0.0098±0.0013	0.0123±0.0011	0.0686±0.0028	0.0145±0.0005	0.0697±0.0023	0.0126±0.0004	0.1338±0.0020	0.0535±0.0019	0.0731±0.0015	0.0270±0.0006
myo-Inositol	nd	nd	nd	nd	nd	nd	nd	nd	0.2548±0.0112	0.1789±0.0149	0.2687±0.0068	0.1651±0.0095
sn-Glycero-3-phosphocholine	nd	nd	nd	nd	nd	nd	nd	nd	0.0804±0.0053	0.1538±0.0092	0.2480±0.0037	0.2662±0.0085
beta-Alanine	nd	nd	nd	nd	nd	nd	nd	nd	0.0425±0.0095	0.0544±0.0028	nd	nd
pi-Methylhistidine	0.0389±0.0045	0.0448±0.0025	0.0637±0.0081	0.0327±0.0032	nd	nd	nd	nd	nd	nd	nd	0.0000±0.0000

nd = not detected. All samples were measured in triplicate. Data are expressed as the mean ± standard deviation (n = 5) based on dry weight.
