# Supplementary figures and images for: Organ-Specific Differential NMR-Based Metabonomic Analysis of Soybean [Glycine max (L.) Merr.] Fruit Reveals the Metabolic Shifts and Potential Protection Mechanisms Involved in Field Mold Infection
Source: Front Plant Sci. 2017 Apr 25;8:508. doi: 10.3389/fpls.2017.00508 (PMC5404178; doi:10.3389/fpls.2017.00508)

## Slide 1
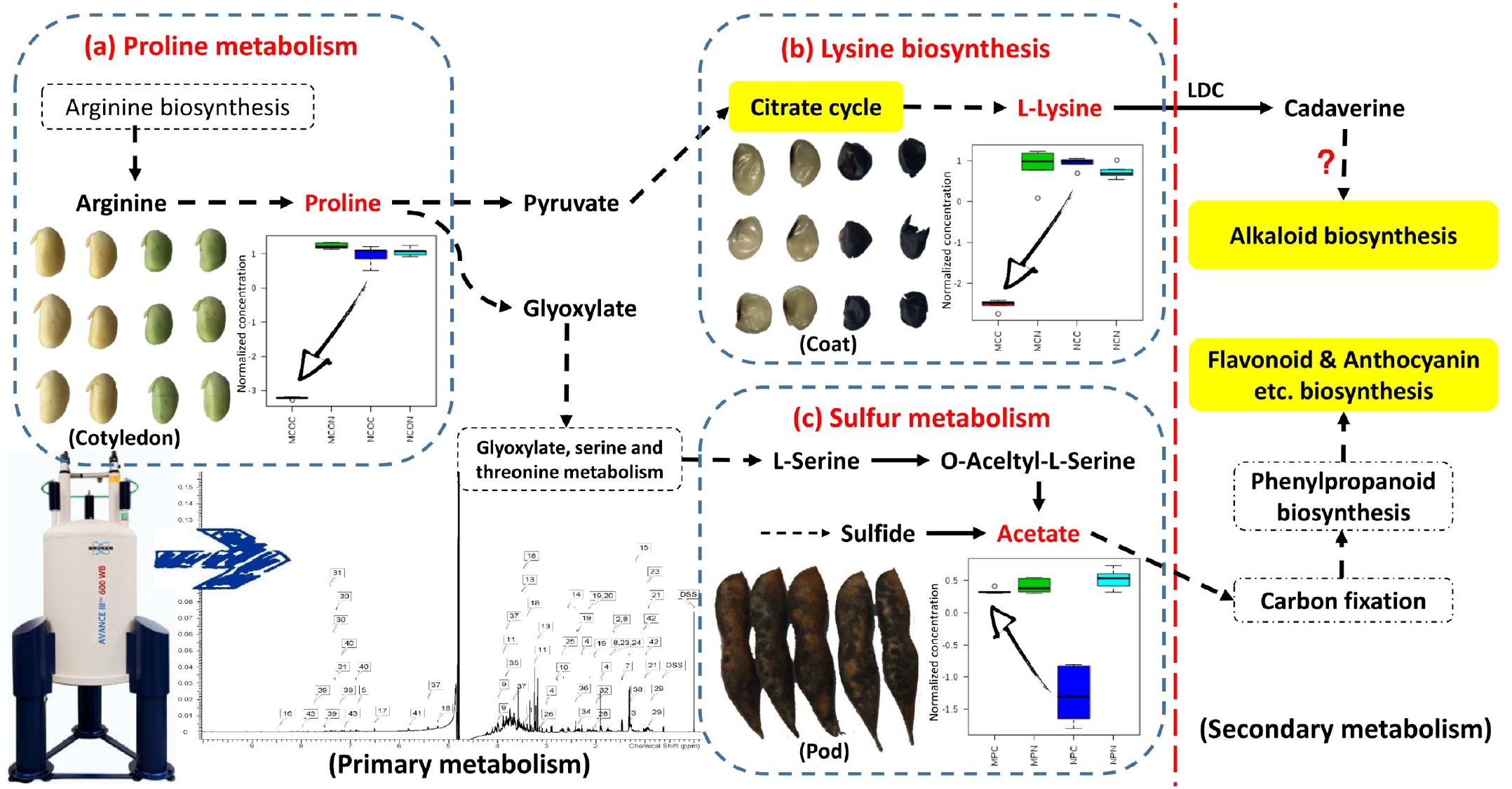

Supplement: Supplementary file 3 [file Presentation1.PPTX]
