# Supplementary material for: Organ-Specific Differential NMR-Based Metabonomic Analysis of Soybean [Glycine max (L.) Merr.] Fruit Reveals the Metabolic Shifts and Potential Protection Mechanisms Involved in Field Mold Infection
Source: Front Plant Sci. 2017 Apr 25;8:508. doi: 10.3389/fpls.2017.00508 (PMC5404178; doi:10.3389/fpls.2017.00508)
Supplement: Supplementary file 4 [file Image1.PDF]

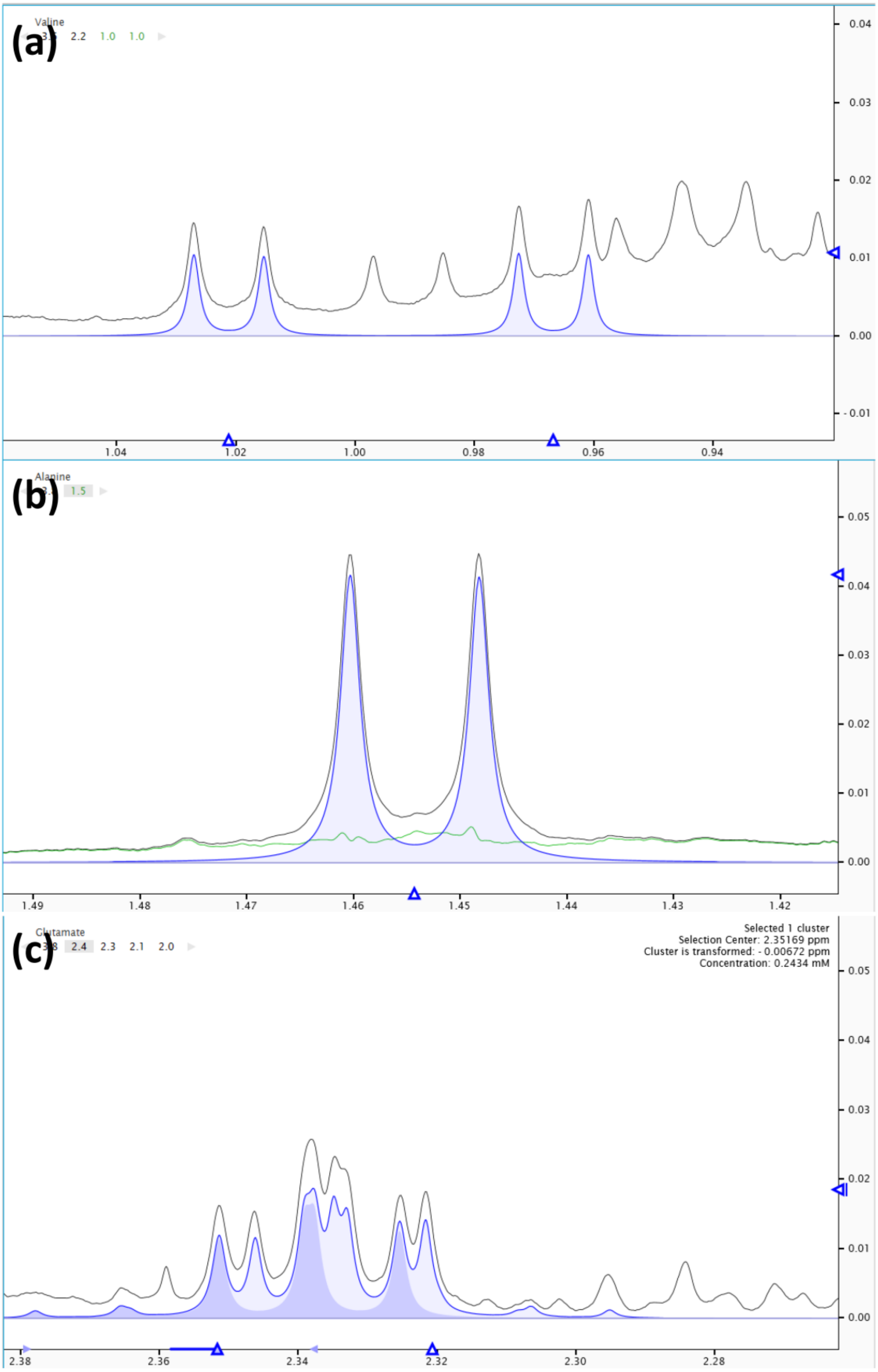

Figure S1. Spectra comparison and metabolite identification using the Chenomx NMR software suite: (**a**) 0.96 ppm, 1.02 ppm; (**b**) 1.45 ppm; (**c**) 2.32 ppm, 2.35 ppm.
