# Supplementary material for: Organ-Specific Differential NMR-Based Metabonomic Analysis of Soybean [Glycine max (L.) Merr.] Fruit Reveals the Metabolic Shifts and Potential Protection Mechanisms Involved in Field Mold Infection
Source: Front Plant Sci. 2017 Apr 25;8:508. doi: 10.3389/fpls.2017.00508 (PMC5404178; doi:10.3389/fpls.2017.00508)
Supplement: Supplementary file 5 [file Image2.PDF]

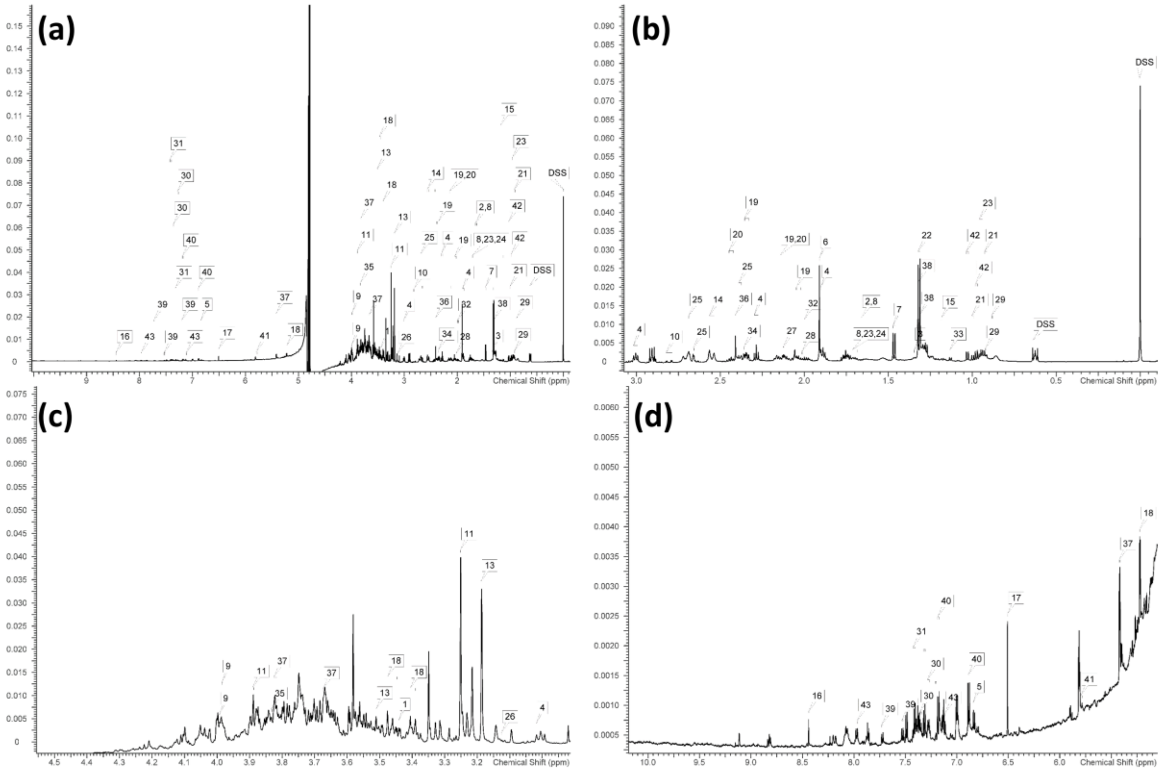

No.	Compound	No.	Compound	No.	Compound	No.	Compound
1	1,3-Dimethylurate	12	Carnitine	23	Leucine	34	Pyruvate
2	2-Aminoadipate	13	Choline	24	Lysine	35	Serine
3	2-Hydroxyisobutyrate	14	Citrate	25	Malate	36	Succinate
4	4-Aminobutyrate	15	Ethanol	26	Malonate	37	Sucrose
5	4-Hydroxyphenylacetate	16	Formate	27	Methionine	38	Threonine
6	Acetate	17	Fumarate	28	N-Acetylglutamate	39	Tryptophan
7	Alanine	18	Glucose	29	Pantothenate	40	Tyrosine
8	Arginine	19	Glutamate	30	Phenylacetate	41	Uracil
9	Asparagine	20	Glutamine	31	Phenylalanine	42	Valine
10	Aspartate	21	Isoleucine	32	Proline	43	pi-Methylhistidine
11	Betaine	22	Lactate	33	Propylene glycol	44	Epicatechin

Figure S2. Typical ^{1}H-NMR spectra of a 50% methanol soybean extract and main metabolite annotations: (a) 0-10 ppm; (b) 0-3 ppm; (c) 3-5 ppm; (d) 5-10 ppm.
