# Supplementary material for: Organ-Specific Differential NMR-Based Metabonomic Analysis of Soybean [Glycine max (L.) Merr.] Fruit Reveals the Metabolic Shifts and Potential Protection Mechanisms Involved in Field Mold Infection
Source: Front Plant Sci. 2017 Apr 25;8:508. doi: 10.3389/fpls.2017.00508 (PMC5404178; doi:10.3389/fpls.2017.00508)
Supplement: Supplementary file 6 [file Image3.PDF]

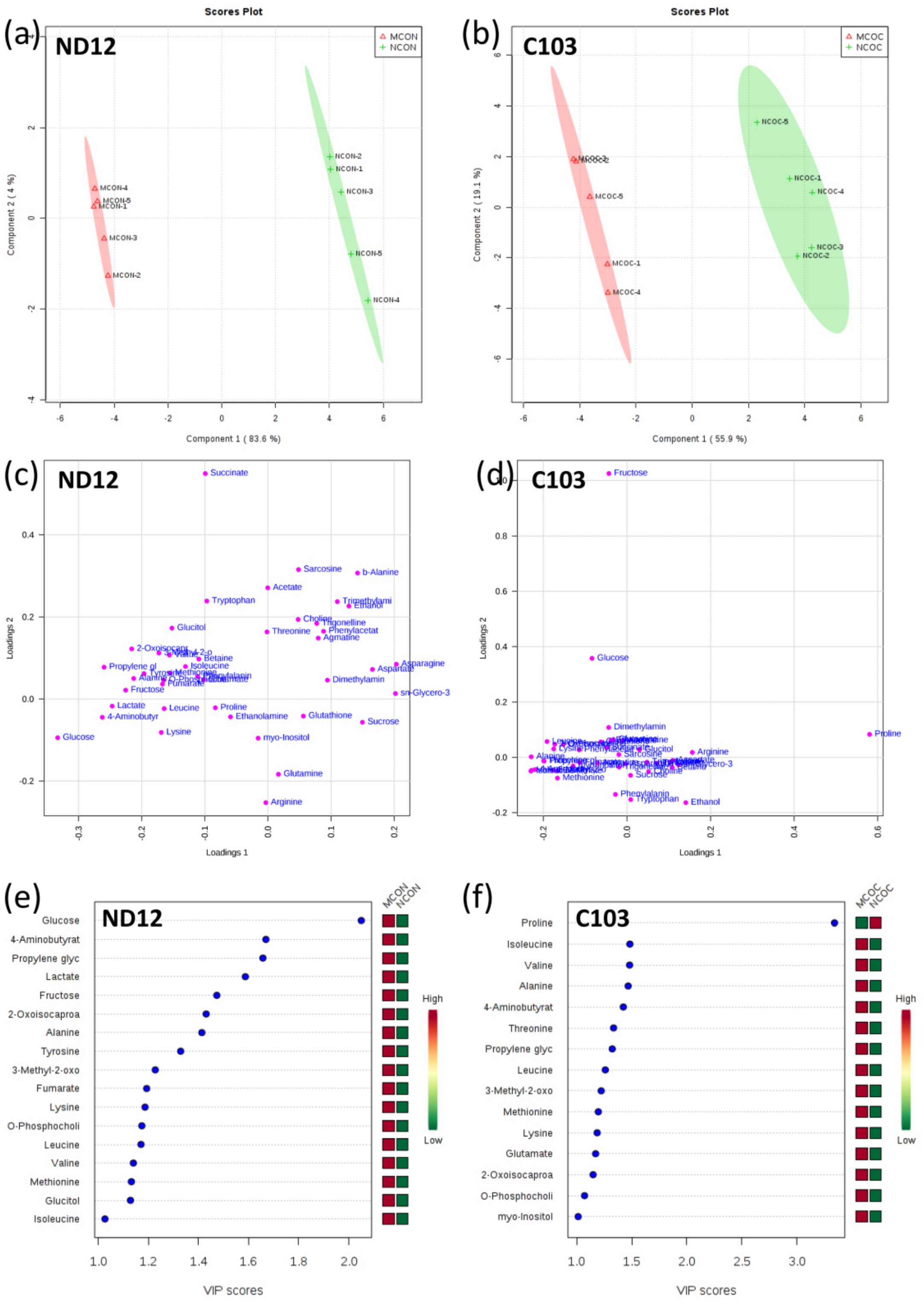

Figure S3. (**a**) PLS-DA score plot of soybean cotyledons of variety ND12 classified as susceptible based on infection status. (**b**) PLS-DA score plot of soybean cotyledons of variety C103 classified as resistant based on infection status. (**c**) Scatter plot of loadings corresponding to panel a. (**d**) Scatter plot of loadings corresponding to panel b. (**e**) Important features identified by PLS-DA corresponding to panel a. (**f**) Important features identified by PLS-DA corresponding to panel b. The colored boxes on the right indicate the relative concentrations of the corresponding metabolites in each group.
