# Supplementary material for: Organ-Specific Differential NMR-Based Metabonomic Analysis of Soybean [Glycine max (L.) Merr.] Fruit Reveals the Metabolic Shifts and Potential Protection Mechanisms Involved in Field Mold Infection
Source: Front Plant Sci. 2017 Apr 25;8:508. doi: 10.3389/fpls.2017.00508 (PMC5404178; doi:10.3389/fpls.2017.00508)
Supplement: Supplementary file 9 [file Image6.PDF]

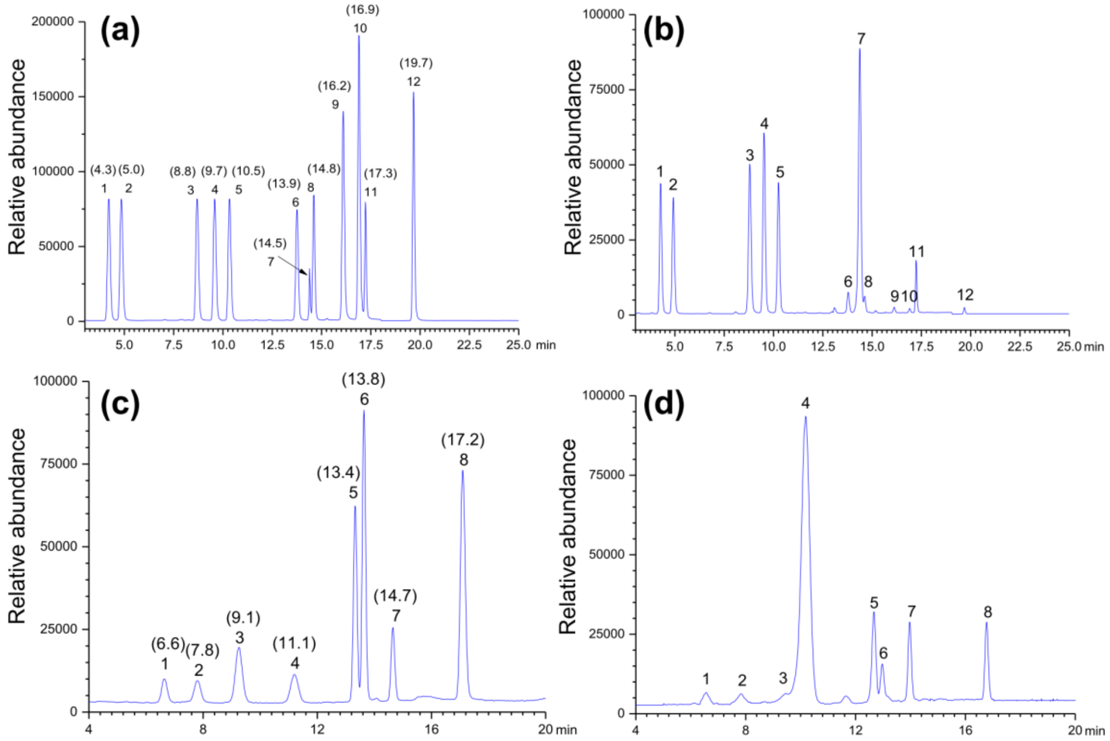

Figure S6. Typical selected ion chromatograms (SIC) of a standard isoflavone mixture (**a**) and black soybean extract (**b**). Assignment of peaks: 1-daidzin (DG), 2-glycitin (GLG), 3-genistin (GEG), 4-malonyldaidzin (MD), 5-malonylglycitin (MGL), 6-acetyldaidzin (AD), 7-malonylgenistin (MG), 8-acetylgenistin (AG), 9-daidzein (DE), 10-glycitein (GLE), 11-acetylglycitin (AGL) and 12-genistein (GE). Typical SICs of a standard anthocyanin mixture (**c**) and black soybean extract (**d**). Assignment of peaks: 1-delphinidin-3-galactoside (DEA), 2-delphinidin-3-glucoside (DEL), 3-cyanidin-3-galactoside (CYA), 4-cyanidin-3-glucoside (CYL), 5-petunidin-3-glucoside (PET), 6-pelargonidin-3-glucoside (PEL), 7-peonidin-3-glucoside (PEO), and 8-cyanidin chloride (CYC).
